# Supplementary material for: Quality assessment and umbrella review of systematic reviews about dance for people with Parkinson’s disease
Source: PLoS One. 2024 Dec 31;19(12):e0311003. doi: 10.1371/journal.pone.0311003 (PMC11687919; doi:10.1371/journal.pone.0311003)
Supplement: S1 File — (DOCX) [file pone.0311003.s002.docx]

**Supplementary Material 1.** Database Search Strategies

**PUBMED**

***All fields**

**#1 OR #2 OR #3 OR #4 OR #5 OR #6 OR #7 OR #8 OR #9 OR #10 OR #11 OR #12 OR #13 OR #14 OR #15 OR #16 OR #17 OR #18 OR #19 OR #20 OR #21 OR #22 OR #23 OR #24 OR #25 OR #26 OR #27 OR #28 OR #29 OR #30 OR #31 OR #32 OR #33 OR #34 OR #35 OR #36 OR #37 OR #38 OR #39 OR #40 => #41**

Dancing [Mesh]

Dance

Ballet

Square Dance

Dance, Square

Hip-Hop Dance

Dance, Hip-Hop

Hip Hop Dance

Jazz Dance

Dance, Jazz

Tap Dance

Dance, Tap

Modern Dance

Dance, Modern

Salsa Dancing

Dancing, Salsa

Line Dancing

Dancing, Line

Dance therapy [Mesh]

Therapy, Dance

Dance Therapies

Therapies, Dance

Dance therap*

Dance intervention

Samba dance

Argentine tango

American ballroom

Tango dance

Waltz dance

Cha-cha dance

Popping dance

Mambo dance

Flamenco dance

Polka dance

Swing dance

Quickstep dance

Forro dance

Dance-based

Dance Parkinsons

Dance PD

**#42 OR #43 OR #44 OR #45 OR #46 OR #47 OR #48 OR #49 OR #50 OR #51 OR #52 OR #53 => #54**

Parkinson disease [Mesh]

Parkinson's Disease

Idiopathic Parkinson Disease

Idiopathic Parkinson's Disease

Lewy Body Parkinson Disease

Lewy Body Parkinson's Disease

Paralysis Agitans

Parkinson Disease, Idiopathic

Parkinson’s Disease, Idiopathic

Parkinson's Disease, Lewy Body

Parkinsonism, Primary

Primary Parkinsonism

**#55 OR #56 OR #57 OR #58 OR #59 OR #60 OR #61 OR #62 OR #63 OR #64 OR #65 => #66**

Review [Publication Type]

Review, Multicase

Review, Academic

Review of reported Cases

Review Literature

Systematic Review [Publication Type]

Meta-Analysis [Publication Type]

Meta Analysis

Meta Analys*

Overview

Systematic Reviews as Topic

**MEDLINE (EBSCO)**

***All fields**

**#S1 OR #S2 OR #S3 OR #S4 => #S5**

Dancing [Mesh]

Dance

Ballet

Square Dance

Dance, Square

Hip-Hop Dance

Dance, Hip-Hop

Hip Hop Dance

Jazz Dance

Dance, Jazz

Tap Dance

Dance, Tap

Modern Dance

Dance, Modern

Salsa Dancing

Dancing, Salsa

Line Dancing

Dancing, Line

Dance therapy

Therapy, Dance

Dance Therapies

Therapies, Dance

Dance therap*

Dance intervention

Samba dance

Argentine tango

American ballroom

Tango dance

Waltz dance

Cha-cha dance

Popping dance

Mambo dance

Flamenco dance

Polka dance

Swing dance

Quickstep dance

Forro dance

Dance-based

Dance Parkinsons

Dance PD

**#S6**

Parkinson disease

Parkinson's Disease

Idiopathic Parkinson Disease

Idiopathic Parkinson's Disease

Lewy Body Parkinson Disease

Lewy Body Parkinson's Disease

Paralysis Agitans

Parkinson Disease, Idiopathic

Parkinson’s Disease, Idiopathic

Parkinson's Disease, Lewy Body

Parkinsonism, Primary

Primary Parkinsonism

**#S7**

Review

Review, Multicase

Review, Academic

Review of reported Cases

Review Literature

Systematic Review

Meta-Analysis

Meta Analysis

Meta Analys*

Overview

Systematic Reviews as Topic

**CINAHL (EBSCO)**

***All fields**

**#S1 OR #S2 OR #S3 OR #S4 => Agrupated as #S5**

Dancing

Dance

Ballet

Square Dance

Dance, Square

Hip-Hop Dance

Dance, Hip-Hop

Hip Hop Dance

Jazz Dance

Dance, Jazz

Tap Dance

Dance, Tap

Modern Dance

Dance, Modern

Salsa Dancing

Dancing, Salsa

Line Dancing

Dancing, Line

Dance therapy

Therapy, Dance

Dance Therapies

Therapies, Dance

Dance therap*

Dance intervention

Samba dance

Argentine tango

American ballroom

Tango dance

Waltz dance

Cha-cha dance

Popping dance

Mambo dance

Flamenco dance

Polka dance

Swing dance

Quickstep dance

Forro dance

Dance-based

Dance Parkinsons

Dance PD

**#S6**

Parkinson disease

Parkinson's Disease

Idiopathic Parkinson Disease

Idiopathic Parkinson's Disease

Lewy Body Parkinson Disease

Lewy Body Parkinson's Disease

Paralysis Agitans

Parkinson Disease, Idiopathic

Parkinson’s Disease, Idiopathic

Parkinson's Disease, Lewy Body

Parkinsonism, Primary

Primary Parkinsonism

**#S7**

Review

Review, Multicase

Review, Academic

Review of reported Cases

Review Literature

Systematic Review

Meta-Analysis

Meta Analysis

Meta Analys*

Overview

Systematic Reviews as Topic

**SPORT DISCUSS (EBSCO)**

**#S1 OR #S2 OR #S3 OR #S4 => Agrupated as #S5**

Dancing

Dance

Ballet

Square Dance

Dance, Square

Hip-Hop Dance

Dance, Hip-Hop

Hip Hop Dance

Jazz Dance

Dance, Jazz

Tap Dance

Dance, Tap

Modern Dance

Dance, Modern

Salsa Dancing

Dancing, Salsa

Line Dancing

Dancing, Line

Dance therapy

Therapy, Dance

Dance Therapies

Therapies, Dance

Dance therap*

Dance intervention

Samba dance

Argentine tango

American ballroom

Tango dance

Waltz dance

Cha-cha dance

Popping dance

Mambo dance

Flamenco dance

Polka dance

Swing dance

Quickstep dance

Forro dance

Dance-based

Dance Parkinsons

Dance PD

**#S6**

Parkinson disease

Parkinson's Disease

Idiopathic Parkinson Disease

Idiopathic Parkinson's Disease

Lewy Body Parkinson Disease

Lewy Body Parkinson's Disease

Paralysis Agitans

Parkinson Disease, Idiopathic

Parkinson’s Disease, Idiopathic

Parkinson's Disease, Lewy Body

Parkinsonism, Primary

Primary Parkinsonism

**#S7**

Review

Review, Multicase

Review, Academic

Review of reported Cases

Review Literature

Systematic Review

Meta-Analysis

Meta Analysis

Meta Analys*

Overview

Systematic Reviews as Topic

**EMBASE (Elsevier)**

**INTERVENTION *(OR)***

Dancing

Dance

Ballet

Square Dance

Dance, Square

Hip-Hop Dance

Dance, Hip-Hop

Hip Hop Dance

Jazz Dance

Dance, Jazz

Tap Dance

Dance, Tap

Modern Dance

Dance, Modern

Salsa Dancing

Dancing, Salsa

Line Dancing

Dancing, Line

Dance therapy

Therapy, Dance

Dance Therapies

Therapies, Dance

Dance therap*

Dance intervention

Samba dance

Argentine tango

American ballroom

Tango dance

Waltz dance

Cha-cha dance

Popping dance

Mambo dance

Flamenco dance

Polka dance

Swing dance

Quickstep dance

Forro dance

Dance-based

Dance Parkinsons

Dance PD

**AND**

**POPULATION *(OR)***

Parkinson disease

parkinson`s Disease

Idiopathic Parkinson Disease

Idiopathic parkinson`s Disease

Lewy Body Parkinson Disease

Lewy Body parkinson`s Disease

Paralysis Agitans

Parkinson Disease, Idiopathic

parkinson`s Disease, Idiopathic

parkinson`s Disease, Lewy Body

Parkinsonism, Primary

Primary Parkinsonism

**AND**

**TYPE *(OR)***

Review

Review, Multicase

Review, Academic

Review of reported Cases

Review Literature

Systematic Review

Meta-Analysis

Meta Analysis

Meta Analys*

Overview

Systematic Reviews as Topic

**Overall Seach:** ('parkinson disease'/exp OR 'lewy bodies of parkinson disease' OR 'lewy bodies of parkinson`s disease' OR 'lewy bodies of parkinsons disease' OR 'lewy body parkinsons disease' OR 'parkinson dementia complex' OR 'parkinson disease' OR 'parkinsons disease' OR 'idiopathic parkinsonism' OR 'parkinson`s disease' OR 'idiopathic parkinson disease'/exp OR 'idiopathic parkinson`s disease' OR 'lewy body parkinson disease' OR 'lewy body parkinson`s disease' OR 'paralysis agitans' OR 'parkinson`s disease, idiopathic' OR 'parkinson disease, idiopathic' OR 'parkinson`s disease, lewy body' OR 'parkinsonism, primary' OR 'primary parkinsonism') **AND** (dancing OR 'dancing'/exp OR ballet OR 'square dance' OR 'dance, square' OR 'hip-hop dance' OR 'dance, hip-hop' OR 'hip hop dance' OR 'jazz dance' OR 'dance, jazz' OR 'tap dance' OR 'dance, tap' OR 'modern dance' OR 'dance, modern' OR 'salsa dancing' OR 'dancing, salsa' OR 'line dancing' OR 'dancing, line' OR 'dance therapy' OR 'therapy, dance' OR 'dance therapies' OR 'therapies, dance' OR 'dance therap*' OR 'dance intervention' OR 'samba dance' OR 'argentine tango' OR 'american ballroom' OR 'tango dance' OR 'waltz dance' OR 'cha-cha dance' OR 'popping dance' OR 'mambo dance' OR 'flamenco dance' OR 'polka dance' OR 'swing dance' OR 'quickstep dance' OR 'forro dance' OR 'dance based' OR 'dance parkinsons' OR 'dance pd') **AND** (review OR 'review, multicase' OR 'review, academic' OR 'review of reported cases' OR 'review literature' OR 'systematic review' OR 'meta analysis' OR 'meta analys*' OR overview OR 'systematic reviews as topic')

**CENTRAL (Cochrane Library)**

**#1** (parkinson disease OR lewy bodies of parkinson disease OR lewy bodies of parkinson's disease OR lewy bodies of parkinsons disease OR lewy body parkinsons disease OR parkinson dementia complex OR parkinson disease OR parkinsons disease OR idiopathic parkinsonism OR parkinson's disease OR idiopathic parkinson disease OR idiopathic parkinson's disease OR lewy body parkinson disease OR lewy body parkinson's disease OR paralysis agitans OR parkinson's disease, idiopathic OR parkinson disease, idiopathic OR parkinson's disease, lewy body OR parkinsonism, primary OR primary parkinsonism) (Word variations have been searched)

**#2** dancing OR dancing OR ballet OR square dance OR dance,square OR hip-hop dance OR dance, hip-hop OR hip hop dance OR jazz dance OR dance, jazz OR tap dance OR dance, tap OR modern dance OR dance, modern OR salsa dancing OR dancing, salsa OR line dancing OR dancing, line OR dance therapy OR therapy, dance OR dance therapies OR therapies, dance OR dance therap* OR dance intervention OR samba dance OR argentine tango OR american ballroom OR tango dance OR waltz dance OR cha-cha dance OR popping dance OR mambo dance OR flamenco dance OR polka dance OR swing dance OR quickstep dance OR forro dance OR dance based OR dance parkinsons OR dance pd

**#3** review OR review, multicase OR review, academic OR review of reported cases OR review literature OR systematic review OR meta analysis OR meta analys* OR overview OR systematic reviews as topic

**#4** #1 AND #2 AND #3

**PEDRO (Cochrane Library)**

Search 1: dance AND parkinson AND systematic review

Search 2: dancing AND parkinson AND systematic review

**LILACS**

Search 1: dance AND parkinson AND review

Search 2: dancing AND parkinson AND review

**SCIELO**

Search 1: dance AND parkinson AND review

Search 2: dancing AND parkinson AND review

**APA PsycNet (APA Pshychinfo)**

Search 1: dance AND parkinson AND review

Search 2: dancing AND parkinson AND review

**SCOPUS**

dance AND parkinson AND review

**AMED (Allied and Complementary Medicine Database)**

dance AND parkinson AND review
