# Supplementary material for: Quality assessment and umbrella review of systematic reviews about dance for people with Parkinson’s disease
Source: PLoS One. 2024 Dec 31;19(12):e0311003. doi: 10.1371/journal.pone.0311003 (PMC11687919; doi:10.1371/journal.pone.0311003)
Supplement: S1 Table — (DOCX) [file pone.0311003.s003.docx]

| Supplementary Material 2. | | | | | | | | | |
| --- | --- | --- | --- | --- | --- | --- | --- | --- | --- |
| **Checklists of Included Reviews with Meta-analyses** | | | | | | | | | |
| **Authors** | **Year** | **Title** | **Cochrane Handbook** | **Cochrane**  **or**  **PROSPERO**  **Prior Registration** | **PRISMA Guidelines** | **GRADE** | **Languages**  **Included** | **Search**  **From**  **Inception**  **to** | **Identification of Studies via Databases, Registries, and other Methods** |
| Wei-Hsin Cheng, et al. | 2024 | The effect of dance on mental health and quality of life of people with Parkinson’s disease: A systematic review and three-level meta-analysis | Yes | No | Yes | No | English | Dec 2022 | Databases: PubMed, Cochrane Library, and Embase Other methods: No |
| Ernst M., et al. | 2024 | Physical exercise for people with Parkinson’s disease: a systematic review and network meta-analysis (Cochrane Review) | Yes | Yes | Yes | Yes | No  restrictions | May  2021 | Databases: CENTRAL, MEDLINE, Embase, CINAHL, SPORTDiscus, AMED, REHABDATA, PEDro, EU Clinical Trials Register, WHO, ClinicalTrials.gov, ISRCTN registry.  Other methods: trial registries, conference proceedings, and reference list |
| Donida G., et al. | 2023 | Efectos de la danza sobre el equilibrio de personas con la enfermedad de Parkinson: una revisión sistemática con metaanálisis | Yes | Yes | Yes | No | English, Portuguese, and Spanish | April  2020 | MEDLINE, LILACS, Cochrane, PEDro, PsycINFO and Google Scholar.  Other methods: No |
| Caroline Simpkins & Yang | 2023 | Do dance style and intervention duration matter in improving balance among people with Parkinson’s disease? A Systematic review with Meta-analysis | No | No | No | No | English | Jun  2022 | Databases: PubMed, PsycINFO, Cochrane Library, CINAHL, and MEDLINE  Other methods: No |
| Meiqi Zhang, et al. | 2023 | Exercise sustains motor function in Parkinson's disease: Evidence from 109 randomized controlled trials on over 4,600 patients | No | Yes | Yes | No | English | Aug  2021 | Databases: PubMed, Embase, and MEDLINE  Other methods: No |
| Di Wang, et al. | 2023 | Effectiveness of different exercises in improving postural balance among Parkinson’s disease patients: a systematic review and network meta-analysis | Yes | Yes | Yes | No | No restrictions | Mat 2023 | Databases: PubMed, Embase, PsycINFO, Cochrane Central Register of Controlled Trials (CENTRAL), and Web of Science Other methods: No |
| Hayam Mahmoud Mahmoud, et al. | 2023 | Effect of dancing on freezing of gait in patients with Parkinson’s disease: A systematic review and meta-analysis | No | Yes | Yes | Yes | English | Apr 2022 | Databases: PubMed, MEDLINE, Cochrane Library, WOS, Wiley online library, and EBSCO Other methods: No |
| Shenglan He, et al. | 2023 | Whether mindfulness-guided therapy can be a new direction for the rehabilitation of patients with Parkinson’s disease: a network meta-analysis of non-pharmacological alternative motor -/sensory-based interventions | No | No  (Registered in  Inplasy) | Yes | No | English | Oct  2022 | Databases: PubMed, Cochrane Library, Embase, WOS, and EBSCO CINAHL Other methods: No |
| Patrícia Lorenzo-García, et al. | 2023 | Effects of physical exercise interventions on balance, postural stability and general mobility in Parkinson’s disease: a network meta-analysis | Yes | Yes | Yea | Yes | English and Spanish | Aug 2022 | Databases: PubMed, Cochrane Central Register of Controlled Trials, and WOS Other methods:references from previous systematic reviews, meta-analyses, and suitable articles were reviewed |
| Sara Mohamed Hasan, et al. | 2022 | Efficacy of dance for Parkinson’s disease: a pooled analysis of 372 patients | Yes | No | Yes | No | English | Apr  2020 | Databases: MEDLINE (Pubmed), Scopus, WOS, and Cochrane CENTRAL.  Other methods: No |
| Claire Chrysanthi Karpodini, et al. | 2022 | Rhythmic cueing, dance, resistance training, and Parkinson's disease: A systematic review and meta-analysis | Yes | Yes | Yes | Yes | No  restrictions | Jun  2020 | Databases: Cochrane CENTRAL, MEDLINE (PubMed), and SPORTDisc.  Other methods: No |
| Chun-Lan Yang, et al. | 2022 | Effects and parameters of community-based exercise on motor symptoms in Parkinson’s disease: a meta-analysis | Yes | Yes | Yes | No | English | Apr  2022 | Databases: PEDro, MEDLINE (PubMed), Cochrane CENTRAL, Scopus, Embase, and WOS.  Other methods: references list, and Google Scholar |
| Rustem Mustafaoglu, et al. | 2022 | Which type of mind–body exercise is most effective in improving functional performance and quality of life in patients with Parkinson's disease? A systematic review with network meta-analysis | Yes | Yes | Yes | No | English | Dec  2021 | Databases: MEDLINE (PubMed), Cochrane CENTRAL, and WOS.  Other methods: Google Scholar and reference list |
| Patricia Lorenzo-García, et al. | 2022 | Physical Exercise Interventions on Quality of Life in Parkinson’s Disease: A Network Meta-analysis | No | Yes | Yes | Yes | No  restrictions | Jan  2022 | Databases: MEDLINE (PubMed), PEDro, Cochrane CENTRAL, and WOS.  Other methods: previous systematic reviews and meta-analyses on the topic and the reference list |
| Lina Goh, et al. | 2022 | The effect of rehabilitation interventions on freezing of gait in people with Parkinson’s disease is unclear: a systematic review and meta-analyses | No | Yes | No | Yes | No  restrictions | Jun  2021 | Databases: CINAHL, Cochrane CENTRAL, EMBASE, MEDLINE, OTSeeker, PEDro, and Scopus.  Other methods: reference list |
| Yong Yang, et al. | 2022 | Efficacy and evaluation of therapeutic exercises on adults with Parkinson’s disease: a systematic review and network meta-analysis | No | Yes | Yes | No | No  restrictions | Jun  2022 | Databases: PubMed, MEDLINE, Embase, PsycINFO, Cochrane CENTRAL, WOS, and, CNKI.  Other methods: reference list |
| Yuxin Wang, et al. | 2022 | Efficacy of non-pharmacological interventions for depression in individuals with Parkinson’s disease: A systematic review and network meta-analysis | No | No | No | No | No  restrictions | Apr  2022 | Databases: PubMed, Embase, Cochrane CENTRAL, PsycINFO, CNKI, and Wanfang.  Other methods: gray literature and reference list |
| Zikang Hao, et al. | 2022 | Effects of Ten Different Exercise Interventions on Motor Function in Parkinson’s Disease Patients—A Network Meta-Analysis of Randomized Controlled Trials | No | No | Yes | No | No  restrictions | Apr  2022 | Databases: PubMed, Embase, Cochrane Library, WOS, and CNKI.  Other Methods: No |
| Li‐li Wang, et al. | 2022 | Effects of dance therapy on non‐motor symptoms in patients with Parkinson’s disease: a systematic review and meta‐analysis | No | No | Yes | No | English | Oct  2021 | Databases: PubMed, Web of Science, The Cochrane Library, Embase, and Science Direct.  Other methods: Reference list |
| Celia Alvarez-Bueno, et al. | 2021 | Effect of exercise on motor symptoms in patients with Parkinson’s Disease: a network meta-analysis | No | Yes | Yes | Yes | No  restrictions | Apr  2021 | Databases: MEDLINE, WOS, Scopus, and Cochrane CENTRAL.  Other methods: systematic reviews and metaanalyses, and reference list |
| Sophia Rasheeqa Ismail, et al. | 2021 | Evidence of disease severity, cognitive and physical outcomes of dance interventions for persons with Parkinson’s Disease: a systematic review and meta-analysis | Yes | Yes | Yes | Yes | No  restrictions | Jun  2021 | Databases: MEDLINE, EMBASE, Cochrane CENTRAL, WHO.  Other methods: reference list, lists of retrieved articles published, unpublished or ongoing studies |
| Danique L. M. Radder, et al. | 2020 | Physiotherapy in Parkinson’s Disease: A Meta-Analysis of Present Treatment Modalities | Yes | No | No | Yes | English | Jun  2020 | Databases: Pubmed (Medline), CINAHL, Embase, and WOS.  Other methods: reference list Full-text articles, published abstracts, and conference proceedings. |
| Maxwell Barnish & Barran | 2020 | A systematic review of active group-based dance, singing, music therapy and theatrical interventions for quality of life, functional communication, speech, motor function and cognitive status in people with Parkinson’s disease | No | No | Yes | No | English | Feb  2020 | Databases: PsycINFO, AMED, CINAHL, EMBASE, and MEDLINE.  Other methods: Google Scholar and reference list |
| Ruben D. Hidalgo-Agudo, et al. | 2020 | Additional Physical Interventions to Conventional Physical Therapy in Parkinson’s Disease: A Systematic Review and Meta-Analysis of Randomized Clinical Trials | Yes | No | Yes | No | No  restrictions | Dec  2017 | Databases: WOS, PubMed, Scopus, Scielo, and PEDro.  Other methods: No |
| Anna M. CarapellottiI, et al. | 2020 | The efficacy of dance for improving motornon-motor symptoms, and quality of life in Parkinson’s disease: A systematic review and meta-analysis | Yes | No | No | No | No  restrictions | Mar  2020 | Databases: MEDLINE, Embase, PsycINFO, CINAHL, and PubMed.  Other methods: No |
| Kui Chen, et al. | 2020 | Effect of Exercise on Quality of Life in Parkinson’s Disease: A Systematic Review and Meta-Analysis | No | Yes | Yes | No | No  restrictions | Aug  2018 | Databases: PubMed, Embase, WOS, Cochrane CENTRAL, Clinical Trials, and ISRCTN registry, ProQuest, E+OS, DART-Europe, and NDLTD, PEDro.  Other methods: reference list |
| Heloisa de Almeida, et al. | 2020 | Effect of Dance on Postural Control in People with Parkinson’s Disease: A Meta-Analysis Review | Yes | Yes | Yes | No | No  restrictions | May  2019 | Databases: MEDLINE, LILACS, Cochrane CENTRAL, PEDro, WOS, Scopus, SPORTDiscus, ScienceDirect, and CINAHL.  Other methods: No |
| Nadeesha Kalyani H. Haputhanthirigea, et al. | 2019 | Effects of Dance on Gait, Cognition, and Dual-Tasking in Parkinson’s Disease: A Systematic Review and Meta-Analysis | Yes | Yes | Yes | No | English | Sep  2017 | Databases: PubMed, Cochrane CENTRAL, Embase, CINAHL, PsycINFO, WOS, and Scopus.  Other methods: No |
| Lijun Tang, et al. | 2019 | The effects of exercise interventions on Parkinson’s disease: A Bayesian network meta-analysis | No | No | No | No | English | Mar  2019 | Databases: Embase, Pubmed and Cochrane Library.  Other methods: No |
| Qi Zhang, et al. | 2019 | Effects of dance therapy on cognitive and mood symptoms in people with Parkinson's disease: A systematic review and meta-analysis | Yes | No | No | No | No  restrictions | Dec  2018 | Databases: MEDLINE, CINAHL, Embase, and Cochrane CENTRAL.  Other methods: reference list |
| Camila Monteiro Mazzarin, et al. | 2017 | Effects of Dance and of Tai Chi on Functional Mobility, Balance, and Agility in Parkinson’s Disease - A Systematic Review and Meta-analysis | No | No | Yes | No | English | Apr  2015 | Database: PEDro, MEDLINE, Cochrane Library, Bireme, PubMed, Rehabdata, EMBASE, and CINAHL. Other methods: No |
| Marcela Delabary, et al. | 2017 | Effects of dance practice on functional mobility, motor symptoms and quality of life in people with Parkinson’s disease: a systematic review with meta-analysis | No | Yes | No | No | English, Spanish, and Portuguese | Aug  2017 | Databases: MEDLINE, LILACS, SciELO, Cochrane, and PsycINFO.  Other methods: No |
| Kwok Yan Yan, et al. | 2016 | Effects of mind-body exercises on the physiological and psychosocial well-being of individuals with Parkinson’s disease: A systematic review and meta-analysis | Yes | No | Yes | No | English | Jan  2016 | Database: EMBASE, Ovid Medline, Psych Info, and Cochrane Library. Other methods: reference list |
| Désirée Lötzke, et al. | 2015 | Argentine tango in Parkinson’s disease – a systematic review and meta-analysis | No | No | Yes | No | German or English | Jan  2015 | Databases: PubMED, AMED, CAMbase. Other methods: Google Scholar |
| Joanne Shanahan, et al. | 2015 | Dance for People with Parkinson’s Disease: What Is the Evidence Telling Us? | Yes | No | No | No | No  restrictions | Apr  2014 | Databases: MEDLINE, CINAHL, AMED, SPORTDiscus, PubMed, PubMed Central, Sage, and ScienceDirect. Other methods: reference list |
| Priscila A. da Rocha, et al. | 2015 | Complementary physical therapies for movement disorders in Parkinson’s disease: a systematic review | No | No | No | No | English, Spanish, and Portuguese | Not informed | Databases: Medline, Embase, Cinahl, The Cochrane Library, and PEDro. Other methods: Reference list |
| Kathryn Sharp & Hewitt | 2014 | Dance as an intervention for people with Parkinson’s disease: A systematic review and meta-analysis | No | No | Yes | No | No  restrictions | Jan  2014 | Databases: AMED, BNI, CINAHL, EMBASE, HBE, HMIC, MEDLINE, PsychINFO, Cochrane CENTRAL, Web of Knowledge, OTseeker, PEDro, SpeechBITE, PsychBITE, Rehabdata. Other methods: grey literature, conference abstracts and proceedings, hand searching journals and reference list |
| M. J. de Dreu, et al. | 2012 | Rehabilitation, exercise therapy and music in patients with Parkinson’s disease: a meta-analysis of the effects of music-based movement therapy on walking ability, balance and quality of life | No | No | No | No | No  restrictions | Aug  2011 | Databases: PubMed, Embase, Cochrane, Cinahl, and SPORTDiscus. Other methods: No |
| Claire L. Tomlinson, et al. (A) | 2012 | Physiotherapy versus placebo or no intervention in Parkinson's (Cochrane Review) | Yes | Yes | Yes | No | No  restrictions | Dec  2010 | Databases: Medline, Embase, Cumulative Index to Nursing and Allied Health Literature, Web of Science, Allied and Complimentary Medicine Database, REHABDATA, REHADAT, GEROLIT, Latin American and Caribbean Health Sciences Literature, MedCarib, Index Medicus for the Eastern Mediterranean region; Cochrane CENTRAL, CentreWatch Clinical Trials, metaRegister of Controlled Trials, ClinicalTrials.gov, Research Portfolio Online Reporting Tools, PEDro, National Institute of disability and rehabilitation register, National research register. Conference and grey literature databases: Conference Proceedings Citation Index, Dissertation Abstracts, Conference Papers Index, Index to Theses, Electronic Theses Online Service, ProQuest. Other methods: Relevant specific journals, abstract books, conference proceedings, reference lists. |
| Claire L. Tomlinson, et al. (B) | 2012 | Physiotherapy intervention in Parkinson’s disease: systematic review and meta-analysis | Yes | Yes | No | No | No  restrictions | Jan  2012 | Databases: Medline, Embase, Cumulative Index to Nursing and Allied Health Literature, Web of Science, Allied, and Complimentary Medicine Database, REHABDATA, REHADAT, GEROLIT, Latin American and Caribbean Health Sciences Literature, MedCarib, Index Medicus for the Eastern Mediterranean region; Cochrane CENTRAL, CentreWatch Clinical Trials, meta register of Controlled Trials, ClinicalTrials.gov, Research Portfolio Online Reporting Tools, PEDro, National Institute of disability and rehabilitation register, National research register. Conference and grey literature databases: Conference Proceedings Citation Index, Dissertation Abstracts, Conference Papers Index, Index to Theses, Electronic Theses Online Service, ProQuest. Other methods: Relevant specific journals, abstract books, conference proceedings, reference lists. |
| **Checklists of Included Reviews without Meta-analyses** | | | | | | | | | |
| **Authors** | **Year** | **Title** | **Cochrane Handbook** | **Cochrane**  **or**  **PROSPERO**  **Prior Registration** | **PRISMA Guidelines** | **GRADE** | **Languages**  **Included** | **Search**  **From**  **Inception**  **to** | **Identification of Studies via Databases, Registries, and other Methods** |
| Valton Costa, et al. | 2023 | Physical exercise for treating non-motor symptoms assessed by general Parkinson’s disease scales: systematic review and meta-analysis | No | No | Yes | No | English, Portuguese, and Spanish | Dec 2022 | Databases: PubMed, Cochrane Library, Scopus, WOS, Embase, ScienceDirect, and PEDro Other methods: No |
| Raluca-Dana Mot & Almăjan-Guță | 2022 | Dance therapy for Parkinson’s disease: a systematic review | No | No | No | No | English | 2022 | Databases: Pubmed. Other methods: Google |
| Riddhi Dipak Patel & Mans | 2022 | Review: Effect of Supplemental Activities on Motor and Nonmotor Outcomes in the Parkinson’s Population | No | No | No | No | English | 2022 | Databases: PubMed, CINAHL, ScienceDirect, and Galileo/Discover@GeorgiaSouthern. Other  methods: No |
| Cheng-Cheng Wu, et al. | 2022 | Dance movement therapy for neurodegenerative diseases: A systematic review | No | No  (Registered in  Open Science Framework) | No | No | English | Feb , 2022 | Databases: PubMed, WOS, Cochrane library and PEDro. Other methods: No |
| Sara Emmanouilidis, et al. | 2021 | Dance Is an Accessible Physical Activity for People with  Parkinson’s Disease | Yes | No | Yes | No | English | Jun  2020 | Databases: AMED, Cochrane Library, PEDro, CINHAL, PsycINFO, EMBASE and MEDLINE. Other methods: No |
| A. Berti, et al. | 2020 | Argentine tango in the care of Parkinson’s disease: A systematic review and analysis of the intervention | No | Not clear | Yes | No | English | Nov  2019 | Databases: PubMed, CINAHL, EMBASE, PsycINFO, and PEDro. Other methods: No |
| Adijatu Raheem & CasacaCarreira | 2018 | Effects of ballroom dancing in patients with Parkinson`s disease: A systematic review | No | No | Yes | No | No restrictions | 2017 | Databases: PubMed and PEDro. Other methods: No |
| Lorenna Pryscia C. Aguiar, et al. | 2016 | Therapeutic Dancing for Parkinson's Disease | No | No | No | No | English | Sept  2015 | Databases: CINHAL, Medline, Scopus, WOS,  Embase, PEDro, Cochrane Library. Other methods: No. |
| Melanie E. Cusso, et al. | 2016 | The Impact of Physical Activity on Non-Motor Symptoms in Parkinson’s Disease: A systematic Review | No | Yes | Yes | No | Not informed | Jun 2016 | Databases: PubMed, MEDLINE, Ovid,  SportsDiscuss, Scopus, and WOS. Other methods: Reference list |
| Luís A. A. Santos, et al. | 2016 | Effects of dual-task interventions on gait performance of patients with Parkinson’s Disease: A systematic review | No | No | No | No | Not informed | Dec  2015 | Databases: MEDLINE (PubMed), LILACS and SciELO. Other methods: No |
| Rastilav Šumec, et al. | 2015 | Psychological Benefits of Nonpharmacological  Methods Aimed for Improving Balance in Parkinson’s Disease: A Systematic Review | No | No | No | No | Not informed | Feb  2015 | Databases: PubMed, WOS, and EBSCO. Other methods: No |
| Rosalind Mandelbaum & Lo | 2014 | Examining Dance as an Intervention in Parkinson’s Disease: A Systematic Review | No | No | No | No | No restrictions | Oct r 2013 | Databases: PubMed, WOS and Cochrane Library. Other methods: Google Scholar. |
| Danielle K. Murray, et al. | 2014 | The effects of exercise on cognition in Parkinson’s disease: a systematic review | No | No | Yes | Yes | English | Oct 2013 | Databases: PubMed, Web of Knowledge, and EBM Reviews. Other methods: No |
| E. Valverde Guijarro & García | 2012 | Efecto de la danza en los enfermos de Parkinson | No | No | No | No | Spanish and English | Jan 2012 | Databases: Pubmed, Ovid, ScienceDirect, Trip  Database, Web of Knowledge, Scielo, Cisne, and Dialnet. Other methods: Google scholar. |
| Marie-Sophie Kiepe, et al. | 2012 | Effects of dance therapy and ballroom dances on physical and mental illnesses: A systematic review | Yes | No | No | No | No restrictions | Mar 2011 | Databases: MEDLINE and PsycINFO. Other methods: reference lists included in the articles and in journals of art therapies (“The  Arts in Psychotherapy”, “American Journal of Dance Therapy” and the German jornal “Musik-, Tanz- und Kunsttherapie”), dance therapists and professional associations (First European Association of Art Therapies  (BKMT/FEAT) and TanztherapeutInnen Deutschland e.V.). |
| Abbreviations. CNKI (China National Knowledge Infrastructure. Cochrane CENTRAL (Cochrane Central Register of Controlled Trials). GRADE (Grading of Recommendations, Assessment, Development, and Evaluations). PRISMA (Preferred Reporting Items for Systematic Reviews and Meta-Analyses). RCT (randomized controlled trial). WOS (Web of Science). WHO: World Health Organization International Clinical Trials Registry Platform. | | | | | | | | | |
